# Bolus/infusional 5-fluorouracil and folinic acid. A report on two prospective, consecutive phase II studies with 5-fluorouracil dose escalation.

**DOI:** 10.1038/bjc.1998.243

**Published:** 1998-05

**Authors:** M. J. Mackean, J. Cassidy, D. I. Jodrell, J. Paul, N. S. Reed, P. A. Canney, H. Yosef, T. Habeshaw, A. G. Robertson, A. McInnes, C. J. Twelves

**Affiliations:** CRC Department of Medical Oncology, Beatson Oncology Centre, Glasgow, UK.

## Abstract

We have used a relatively new trial methodology, the group sequential design, to prospectively evaluate two dose levels of bolus/infusional 5-fluorouracil (5-FU) and folinic acid in 192 consecutive-patients with advanced colorectal carcinoma. On day 1, all patients received 200 mg m(-2) of folinic acid infusion over 2 h. Cohort A (n = 102 patients) received 500 mg m(-2) 5-FU by i.v. 15-min infusion followed by an infusion of 500 mg m(-2) 5-FU over 22 h. Treatment was repeated on day 2 and further cycles given 2-weekly. After sequential analysis excluded a response rate of over 40%, cohort B (n = 90 patients) received an increased dose of 600 mg m(-2) 5-FU bolus and infusion. Patients had received no prior 5-FU therapy and the two cohorts had similar demographic features. In 179 evaluable patients, the overall response rate was 18% (95% CI 12-24%) with CR of 6% and PR of 12%, with no difference between the two cohorts. Overall median survival was 34 weeks (95% CI 30-39) with no significant difference between cohorts (median survival 32 and 37 weeks in cohort A and B respectively; P = 0.27). On multivariate analysis, poor performance status, elevated initial WBC and alkaline phosphatase and low serum albumin were associated with reduced survival (P < 0.05), and initial raised WBC showed an association with reduced likelihood of response (P = 0.002). Overall toxicity was low with CTC grade 3 mucositis, diarrhoea, nausea or vomiting in < or = 6% of patients and no treatment-related deaths. Significant (grade 3 or above) leucopenia was more common in cohort B than in cohort A (9% and 1% respectively); there were more dose reductions, and the median administered dose intensity was lower in cohort B than in cohort A (89% and 97% respectively; P = 0.006). In this group of relatively unselected patients, we have confirmed a relatively low objective response rate and median survival of 7.8 months with this regimen. There was no significant difference in outcome between the two dose levels but the higher dose of 5-FU was associated with more dose reductions and greater toxicity.


British Journal of Cancer (1998) 77(9), 1480–1486
© 1998 Cancer Research Campaign

### Bolus/infusional 5-fluorouracil and folinic acid. A report on two prospective, consecutive phase II studies with 5-fluorouracil dose escalation

**MJ Mackean[1], J Cassidy[1*], DI Jodrell[1†], J Paul[2], NS Reed[2], PA Canney[2], H Yosef[2], T Habeshaw[2], AG Robertson[2], A McInnes[1] and CJ Twelves[1]**

[1]CRC Department of Medical Oncology, Beatson Oncology Centre, Glasgow; [2]Beatson Oncology Centre, Western Infirmary Unit Trust, Glasgow, UK

**Summary** We have used a relatively new trial methodology, the group sequential design, to prospectively evaluate two dose levels of bolus/infusional 5-fluorouracil (5-FU) and folinic acid in 192 consecutive-patients with advanced colorectal carcinoma. On day 1, all patients received 200 mg $m^{-2}$ of folinic acid infusion over 2 h. Cohort A ($n$ = 102 patients) received 500 mg $m^{-2}$ 5-FU by i.v. 15-min infusion followed by an infusion of 500 mg $m^{-2}$ 5-FU over 22 h. Treatment was repeated on day 2 and further cycles given 2-weekly. After sequential analysis excluded a response rate of over 40%, cohort B ($n$ = 90 patients) received an increased dose of 600 mg $m^{-2}$ 5-FU bolus and infusion. Patients had received no prior 5-FU therapy and the two cohorts had similar demographic features. In 179 evaluable patients, the overall response rate was 18% (95% CI 12–24%) with CR of 6% and PR of 12%, with no difference between the two cohorts. Overall median survival was 34 weeks (95% CI 30–39) with no significant difference between cohorts (median survival 32 and 37 weeks in cohort A and B respectively; $P$ = 0.27). On multivariate analysis, poor performance status, elevated initial WBC and alkaline phosphatase and low serum albumin were associated with reduced survival ($P$ < 0.05), and initial raised WBC showed an association with reduced likelihood of response ($P$ = 0.002). Overall toxicity was low with CTC grade 3 mucositis, diarrhoea, nausea or vomiting in ≤ 6% of patients and no treatment-related deaths. Significant (grade 3 or above) leucopenia was more common in cohort B than in cohort A (9% and 1% respectively); there were more dose reductions, and the median administered dose intensity was lower in cohort B than in cohort A (89% and 97% respectively; $P$ = 0.006). In this group of relatively unselected patients, we have confirmed a relatively low objective response rate and median survival of 7.8 months with this regimen. There was no significant difference in outcome between the two dose levels but the higher dose of 5-FU was associated with more dose reductions and greater toxicity.

**Keywords:** 5-FU; folinic acid; metastatic colorectal carcinoma; group sequential triangular test

Colorectal cancer is second only to bronchial carcinoma as a cause of cancer death in the UK. There are currently 28 000 new cases in the UK each year, and around half of these patients will die within 5 years of diagnosis. A meta-analysis of 5-fluorouracil (5-FU) used alone in a conventional bolus schedule showed a disappointing response rate of 11% (Advanced Colorectal Cancer Meta-analysis Project, 1992).

A major locus of action of 5-FU is the enzyme thymidylate synthase (TS), which is responsible for the formation of dTMP from dUMP. The 5-FU metabolite FdUMP is a potent inhibitor of TS, forming an irreversible ternary complex with TS and the cofactor 5,10-$CH_2$-tetrahydrofolate (5,10-$CH_2$-$FH_4$). Reduced intracellular concentrations of 5,10-$CH_2$-$FH_4$ may therefore limit the formation of the ternary complex and hence limit cytotoxicity of 5-FU. This hypothesis provides the rationale for the use of 5-FU in combination with folinic acid (5-CHO-$FH_4$), which is readily converted to 5,10-$CH_2$-$FH_4$, increasing the formation of the ternary complex. A meta-analysis of trials of 5-FU with folinic acid vs 5-FU alone showed a response rate of 23% for the combination (Advanced Colorectal Cancer Meta-analysis Project, 1992).

One internationally used schedule for the combination of 5-FU and folinic acid was developed by De Gramont et al (1988). They described a 48-h regimen in which a 2-h infusion of folinic acid (200 mg $m^{-2}$) is followed by bolus (300–500 mg $m^{-2}$) 5-FU and then a 22-h infusion (300–500 mg $m^{-2}$) of 5-FU. This schedule is repeated on day 2 and repeated at 2-weekly intervals. In the initial phase II study of 37 patients, there was a response rate of 54% (CI 38–70%), and the regimen was well tolerated with no patients experiencing WHO grade 3 toxicity (De Gramont et al, 1988). A second phase II study in 43 patients confirmed this tolerability but showed a lower response rate of 24% (CI 11–37%) (Johnson et al, 1991). We reported previously a retrospective analysis of this combination using 300–500 mg $m^{-2}$ of 5-FU and found a disappointing response rate of 11% (95% CI 4–18%) in 81 evaluable patients (Jodrell et al, 1994). We noted, however, that patients treated at the highest dose (500 mg $m^{-2}$) of 5-FU had a statistically better median survival compared with those treated at 300–400 mg $m^{-2}$ of 5-FU after adjusting for the effects of other prognostic factors (9 months and 5 months respectively, $P$ = 0.001). In view of this possible effect of 5-FU dose on outcome, we performed a prospective study of this regimen at 500 mg $m^{-2}$ of 5-

*Received 20 December 1996*
*Revised 17 June 1997*
*Accepted 21 October 1997*

*Correspondence to:* MJ Mackean, CRC Department of Medical Oncology, Beatson Oncology Centre, Western Infirmary, Glasgow G11 6NT, UK

**Present address:* ANCHOR (Aberdeen and North Centre for Haematology, Oncology and Radiotherapy), Aberdeen Royal Infirmary, Aberdeen, UK. †*Present address:* ICRF Department of Medical Oncology, Western General Hospital, Edinburgh, UK

**Figure 1** Graphical representation of the triangular test

FU. We used the relatively new approach of a group sequential tringular procedure for response analysis. If the low response rate seen in our retrospective study was confirmed, the dose of 5-FU was to be increased by 20% up to 600 mg m$^{-2}$. The group sequential triangular procedure was to be used again to confirm the response rate at this higher dose level. A secondary aim of the study was to assess toxicity of the regimen at these two dose levels.

### PATIENTS AND METHODS

All patients had histologically proven colorectal cancer with locally advanced, unresectable disease or metastases. Patients with brain metastases or who had received prior intravenous 5-FU chemotherapy (either adjuvant or for advanced disease) were excluded. They were required to have WHO performance status of 0, 1 or 2, with adequate renal function and bone marrow reserve, and bidimensionally measurable disease by clinical examination of soft-tissue metastases or radiological imaging with X-ray, ultrasound, CTScan or magnetic resonance imaging (MRI). Standard WHO response criteria were applied (Miller et al, 1981). Patients were eligible irrespective of derangement of liver biochemistry. Before study entry, all patients had a full clinical examination; WHO performance status recorded; and full blood count, plasma biochemical profile, carcinoembryonic antigen (CEA) and CXR taken. Toxicities during treatment were recorded every 2 weeks by medical staff according to CTC criteria. This prospective study was approved by the local ethics committee.

Treatment was given as an inpatient on days 1 and 2 of a 14-day cycle. On each treatment day, patients first received 200 mg m$^{-2}$ of folinic acid infused in 250 ml of saline over 2 h. This was followed by a 15-min infusion of 5-FU in 100 ml of saline. The same dose of 5-FU was then given by infusion in 500 ml of saline over 22 h. In the first cohort of patients, 500 mg m$^{-2}$ of 5-FU was given as the bolus and infusion. For the second cohort of patients, the 5-FU dose was increased by 20% to 600 mg m$^{-2}$. Treatment was repeated at 14-day intervals providing that toxicities had resolved. Patients who had not recovered had treatment delayed for 1 week. The protocol specified that, in those patients experiencing CTC grade III toxicity, the 5-FU dose was to be reduced to 75% and, for those patients with CTC grade IV toxicity, the 5-FU was given at a 50% dose reduction. There was no dose reduction of folinic acid.

Response to treatment was assessed by clinical examination or radiologically (using the same imaging modality as that used for the initial assessment) after four cycles (8 weeks) of treatment. Tumour markers (CEA) and liver biochemistry tests were not included as measures of objective response. Patients with progressive disease stopped chemotherapy, and those with stable disease or

**Figure 2** Graphical representation of the outcome of the triangular test for both dose cohorts a response continued for a further four cycles before reassessment. After eight cycles, patients with stable disease or a response continued with treatment at 2- or 4-weekly intervals at the physician's discretion.

### Statistical methods

The paper describes the results of two consecutive phase II studies. Both were designed to test the null hypothesis that the real response rate was less than or equal to 25%, similar to the response rate from the meta-analysis of 5-FU with folinic acid (Advanced Colorectal Cancer Meta-analysis Project, 1992) against the alternative that it was greater than this. The response rate was similar to the original result using this 5-FU schedule (De Gramont et al, 1988). In each case, the one-sided significance level for the test was set at 2.5% and the power was set at 97.5% when the true response rate was 40%. Both studies were conducted as group sequential triangular tests (Bellissant et al, 1996). This test involves plotting on the *y*-axis a statistic *Z*, which represents the current difference between the observed response and the response specified in the null hypothesis. On the *x*-axis is plotted the statistic *V*, which represents the cumulative information gathered on all patients so far assessed since the start of the trial. Two lines on the graph delineate a triangular region (Figure 1), the coordinates of which are determined by the power, significance level, null and alternate hypotheses of the test. If at any time the plotted point (*x,y*) falls within this triangular region, the study continues. If the plotted point falls below the lower boundary the null hypothesis is accepted. Alternatively, if the plotted point falls above the upper boundary the null hypothesis is rejected. Here, the statistics *Z* and *V* were calculated and plotted after response information became available for each successive group of ten evaluable patients in the 500 mg $m^{-2}$ study and for each successive group of 20 evaluable patients in the 600 mg $m^{-2}$ study. The group size was increased in the second study to make the workload more practical because of the rapid trial accrual. Both studies were set up and analysed using the package PEST 2.2 (Whitehead and Brunier, 1989) using a transformation of the binomial parameter similar to that in Whitehead (1982).

Overall survival and progression-free survival were calculated from the date of study registration. All eligible patients were included and deaths from all causes have been included in the survival analysis. For progression-free survival, deaths from disease were included, but causes of death unrelated to disease progression were treated as censoring events. Kaplan–Meier estimates (Parmar and Machin, 1995) were used to construct the survival curves. Progression times were adjusted in the analysis to occur at the exact times specified for protocol response assessment (every 8 weeks during chemotherapy and every 12 weeks during follow-up). This was to overcome the problem of overestimating the risk of progression by applying Kaplan–Meier techniques to the unadjusted data (Peto, 1984). Survival curves were stopped when fewer than five patients were at risk. Median follow-up times were calculated using the reverse Kaplan–Meier method.

Prognostic factors for survival were identified by means of Cox's proportional hazards model (Parmar and Machin, 1995) using forward and backward stepwise selection techniques after stratifying for study group. Cox's proportional hazard model was also used for univariate survival comparisons. Prognostic factors for response were identified by logistic regression (Armitage and Berry, 1987), again using forward and backward selection techniques. Ordinal categorical variables were compared using the Mann–Whitney *U*-test (Armitage and Berry, 1987). The proportion of patients responding were compared using Pearson's chi-square test (Armitage and Berry, 1987) with no continuity correction.

**Figure 3** Overall survival of both cohorts

**Figure 4** Progression-free survival of both cohorts

### RESULTS

### Patients (*n* = 206)

Between October 1992 and March 1994, a total of 107 patients were treated at the 500 mg $m^{-2}$ 5-FU dose level (cohort A). Between March 1994 and May 1995, a further 99 patients received the 600 mg $m^{-2}$ 5-FU dose (cohort B). The sequential procedure indicated accrual to both cohorts could have ceased after each had entered 60 evaluable patients (Figure 2). However, because of the time required to confirm response data in these 60 patients and the rapid trial accrual, the study did not close until additional patients had been recruited in each cohort.

Pretreatment patient details for cohorts A and B are shown in Table 1. Two patients had prior chemotherapy with intraperitoneal

© Cancer Research Campaign 1998

**Table 1** Pretreatment patient details

| | Cohort A | Cohort B |
|---|---|---|
| Number of eligible patients | 102 | 90 |
| Age (years) | | |
| Median | 63 | 62 |
| Range | 35–82 | 27–79 |
| Interquartile range | 55–68 | 55–67 |
| Time from initial diagnosis to starting study (weeks) | | |
| Median | 35 | 39 |
| Range | 2–409 | 2–396 |
| Interquartile range | 8–35 | 8–96 |
| Site of primary tumour [% (*n*)] | | |
| Colon | 69 (70) | 60 (54) |
| Rectum | 31 (32) | 40 (36) |
| Performance status[a] [% (*n*)] | | |
| 0 | 32 (32) | 18 (16) |
| 1 | 54 (55) | 67 (60) |
| 2 | 14(14) | 16 (14) |
| Prior treatment [% (*n*)] | | |
| None | 4 (4) | 0 |
| Surgery only | 79 (81) | 74 (67) |
| Surgery and XRT | 16 (16) | 24 (22) |
| Surgery and chemotherapy | 1 (1) | 0 |
| Surgery, XRT and chemotherapy | 0 | 1 (1) |
| Initial alkaline phosphatase[b] [% (*n*)] | | |
| ≤ULN | 10 (10) | 3 (2) |
| >ULN | 51 (50) | 44 (36) |
| >2.5 × ULN | 29 (29) | 28 (23) |
| >5 × ULN | 10 (10) | 25 (20) |
| Initial WBC [% (*n*)] | | |
| ≤8.4 | 49 (50) | 52 (47) |
| >8.5 | 51 (52) | 48 (43) |
| Initial albumin [% (*n*)] | | |
| <LLN | 20 (19) | 16 (13) |
| ≥LLN | 80 (75) | 84 (68) |

[a]$P \geq 0.07$, [b]$P = 0.008$ (Mann–Whitney *U*-test). ULN, upper limit normal; LLN, lower limit normal; XRT, radiotherapy.

**Table 2** Toxicity, i.e. worst CTC grade recorded with any course

| CTC grade | 0 | 1 | 2 | 3 | 4 |
|---|---|---|---|---|---|
| Mucositis (%) | | | | | |
| Cohort A | 61 | 23* | 12 | 4 | 0 |
| Cohort B | 39 | 41* | 12 | 8 | 0 |
| Leucopenia (%) | | | | | |
| Cohort A | 78 | 16 | 7 | 1* | 0* |
| Cohort B | 60 | 10 | 12 | 4* | 4* |
| Diarrhoea (%) | | | | | |
| Cohort A | 62 | 21 | 14 | 4 | 0 |
| Cohort B | 57 | 19 | 18 | 6 | 1 |
| Nausea (%) | | | | | |
| Cohort A | 53 | 34 | 11 | 2 | 0 |
| Cohort B | 50 | 34 | 12 | 3 | 0 |
| Vomiting (%) | | | | | |
| Cohort A | 77 | 11 | 8 | 4 | 0 |
| Cohort B | 77 | 11 | 10 | 2 | 0 |

*$P \geq 0.01$

5-FU and intrahepatic doxorubicin. The two cohorts were not randomized but were generally well matched. There were more patients in cohort A of performance status 0 (32% vs 18%), but this did not reach statistical significance ($P = 0.07$). Patients in cohort A had significantly lower initial alkaline phosphatase levels than those in cohort B ($P = 0.008$). There was no difference in the number of patients with metachronous vs synchronous disease or those with locally advanced vs metastatic disease between the two cohorts. Of the 206 patients entered overall, five in cohort A and nine in cohort B were ineligible and were not analysed further for either toxicity or efficacy ($n$ = 192 eligible). Of these 14 ineligible patients, nine had no measurable disease, two had no histological verification, one had prior 5-FU therapy, one had coexistent Hodgkin's disease and one patient was treated with an inappropriate dose of 5-FU (550 instead of 600 mg $m^{-2}$).

### Efficacy ($n$ = 179)

Of the 192 eligible patients, eight and five patients in cohort A and B, respectively, were not evaluable for response because of the lack of repeat assessment with scans. In the 179 evaluable patients in both cohorts, only ten (6%) achieved a complete response. A further 22 patients (12%) had an objective partial response, giving an overall response rate of 18% (95% CI 12–24%). Seventy-nine patients (44%) had stable disease (SD) and 68 patients (38%) had progressive disease. There was no difference between cohort A and B either in response rate (18% for both; $P = 0.94$) or in frequency of SD (45% and 44% respectively). Raised initial alkaline phosphatase and WBC were associated with lower response rate on univariate analysis ($P = 0.03$ and 0.001 respectively). However, on multivariate analysis, only the initial WBC (≤ 8.5 vs >8.5) was associated with poor response rate ($P = 0.002$). The 93 patients with a WBC of ≤ 8.5 had a response rate of 28% compared with 7% in the 86 patients with a WBC >8.5.

To date, a total of 150 patients have died. In cohort A, there are ten patients still alive and the median follow-up is 110 weeks compared with 32 alive and a median follow-up of 57 weeks for cohort B. The overall median survival was 7.8 months (i.e. 34 weeks, 95% CI 30–39 weeks), with no significant difference between median survival in the two 5-FU cohorts (32 and 37 weeks for cohorts A and B respectively) as shown in Figure 3 ($P = 0.27$).

Figure 4 shows progression-free survival of all patients by 5-FU dose cohort. Median progression-free survival is 16 weeks in both cohorts. The difference in progression-free survival between cohort A and B was not statistically significant with an adjusted progression rate ratio of 0.85 (95% CI 0.62–1.18, $P = 0.34$).

On univariate analyses, the following factors were associated with shorter survival: poor performance status ($P < 0.001$), raised initial alkaline phosphatase ($P < 0.001$), CEA ($P = 0.04$), WBC ($P < 0.001$), bilirubin ($P = 0.007$) or AST ($P = 0.01$) and low haemoglobin ($P = 0.01$) or albumin ($P < 0.001$). On multivariate analysis, low albumin ($P < 0.001$), raised WBC ($P < 0.001$), raised alkaline phosphatase ($P = 0.05$) and poor performance status ($P = 0.03$) retained significance. The forward and backward selection techniques produced the same result for both the Cox and the logistic regression analyses. Adjusting for prognostic factors between the two cohorts, the difference in survival was not statistically significant, with an estimated death rate ratio of 0.77 (95% CI 0.54–1.11, $P = 0.16$).

© Cancer Research Campaign 1998

Table 3 Reasons for dose reductions

| | Cohort A | | Cohort B | |
|---|---|---|---|---|
| Percentage of patients with dose reduction | 17 | | 41 | |
| Total number of cycles reduced | 18 | | 44 | |
| As per protocol – grade 3/4 toxicity<br>Protocol violations – grade 2 toxicity | As per protocol<br>8 | Protocol violation<br>10 | As per protocol<br>15 | Protocol violation<br>29 |
| Reasons for reductions | | | | |
| Haematological | 0 | 2 | 5 | 6 |
| Mucositis | 2 | 4[a] | 3 | 7 |
| Diarrhoea | 2 | 3 | 3 | 8 |
| Nausea/vomiting | 2 | 1 | 1 | 2 |
| Hand–foot syndrome | 2 | 0 | 2 | 8[b] |
| Other | 0 | 0 | 2 | 2 |

[a]One patient with grade 1 mucositis. [b]Two patients with grade 1 hand–foot syndrome. Note, five patients had more than one reason for dose reduction.

### Toxicity ($n$ = 192)

There were no treatment-related deaths. Significant toxicities are shown in Table 2. Significant leucopenia (CTC grade 3 or above) was more common in cohort B than in cohort A (8% and 1% respectively, $P = 0.01$). This was associated with three episodes of neutropenic sepsis (two of which required hospital admission) in two patients in cohort B. No patient experienced grade 3 or 4 thrombocytopenia. In cohort B, 61% of patients experienced some degree of mucositis compared with only 39% of cohort A. This was principally due to a higher incidence of grade 1 mucositis in cohort B ($P = 0.01$). Similarly, grade 1 or 2 alopecia was more common in cohort B (44% vs 21%, $P = 0.001$), but no patient experienced grade 3 alopecia. Clinically significant, i.e. grade 2 or above, hand–foot syndrome was more common in cohort B than cohort A, but this difference was not statistically significant (9% and 5% respectively, $P = 0.27$). Similarly there was no difference in the rate of diarrhoea, nausea and vomiting experienced in both cohorts, with only a small number of patients experiencing grade 3 or 4 toxicity.

### Dose intensity

A median of five cycles was given to patients in both cohorts. Eighty-five per cent of patients (81% and 90% of cohort A and B respectively) stopped treatment at or before eight cycles. The reasons for stopping were: progressive disease (54%); excessive toxicity (9%); death from other unrelated causes (4%); completing eight cycles (11%); and other 'miscellaneous reasons' (22%). Treatment delays were similar for both cohorts with 11% of all cycles delayed in 42% of all patients. Of the 125 cycles delayed, 35 were for haematological toxicity, 25 for non-haematological toxicity, 25 for disease-related conditions and the remaining 40 for mostly administrative reasons, such as public holidays.

There was, however, a significant difference in the median percentage of administered relative to intended dose intensity between cohort A and B (97% and 89% respectively, $P = 0.006$). This was due to more patients in cohort B than cohort A having dose reductions (41% and 17% respectively). In cohort A, 67% of patients received at least 90% of the intended dose intensity of 5-FU compared with only 48% in cohort B. In view of this difference in dose reductions, despite the lack of a statistically significant increase in objective toxicity between the two cohorts, the reasons for the dose reductions in each cohort were examined further. Although some of these dose reductions were not specified by the protocol, in each case they resulted from clinically significant toxicities as shown in Table 3.

The greater number of dose reductions in cohort B mean that there was an increase of only 10% in the administered dose of 5-FU between the two cohorts instead of the 20% that had been intended. Therefore, we also examined whether the actual delivered 5-FU dose intensity influenced response or survival. Dividing the patients into three equal-sized cohorts by the actual 5-FU dose given, i.e. ≤ 948 mg of 5-FU $m^{-2}$ per week ($n = 64$), > 948 up to 1018 mg of 5-FU $m^{-2}$ per week ($n = 65$) and > 1018 mg of 5-FU $m^{-2}$ per week ($n = 63$), there was no association between actual 5-FU dose given and survival, progression-free survival or response, even when stratified for the number of cycles received and prognostic factors (data not shown).

### DISCUSSION

In this prospective dose escalation phase II study of bolus/infusional 5-FU and folinic acid, we have shown, in a relatively unselected group of patients with metastatic or advanced colorectal carcinoma, a response rate of only 18% and a median survival of just 7.8 months. In the retrospective analysis performed at our unit (Jodrell et al, 1994), three different doses of 5-FU from 300–500 mg $m^{-2}$ were used. In that study, we found that the higher 5-FU dose of 500 mg $m^{-2}$ was associated with an improved median survival of 9 months compared with a median survival of 5 months at the lower doses (relative hazard = 0.38, 95% CI 0.21–0.70) after adjusting for the effects of PS, age, PALA, primary site and liver function. The current prospective study has not confirmed an effect of 5-FU dose over 500 mg $m^{-2}$ on outcome in this population of patients.

There are now several reports of this 48-h bolus/infusional 5-FU–folinic acid regimen. A striking feature of these studies is the wide variation in response rates and survival that have been reported. The initial phase II report of this regimen described a response rate of 54% (95% CI 38–70%) and a median survival of 18 months (De Gramont et al, 1988). In a subsequent prospective randomized study (De Gramont et al, 1995), 177 evaluable patients were given this regimen, and the response rate was somewhat lower at 32% (95% CI 25–39), with a median survival of 14 months. In broad agreement with these data, two prospective phase

© Cancer Research Campaign 1998

II trials showed a response rate of 24% (95% CI 11–37%) and 38% (95% CI 28–48%) and median survival of 17.3 and 10.3 months respectively (Johnson et al, 1991; Becouarn et al, 1995). By contrast, the results in the current study are similar to those from two retrospective studies of this regimen, both of which had a response rate of only 11% (95% CI 4–18%) and median survival of 6 months (Jodrell et al, 1994) and 8 months (Hanna et al, 1995).

Patient selection is the most probable explanation for these differences. The patients entered into the formal phase II and III studies (De Gramont et al, 1988; 1995; Johnson et al, 1991; Becouarn et al, 1995) may have differed significantly from those in the current study and the retrospective audits (Jodrell et al, 1994; Hanna et al, 1995). The studies are difficult to compare as, unfortunately, not all have described prognostically important patient characteristics. For example, initial liver biochemistry tests are not widely quoted, although these have been identified as influencing prognosis (Petrelli, 1995). The current study had broad entry criteria, with no limits on liver dysfunction, and it accrued patients from 16 oncologists at the Beatson Oncology Centre, serving most of the population of the West of Scotland. The relatively low response rate and survival in the current study, in line with that of the retrospective studies (Jodrell et al, 1994; Hanna et al, 1995) probably more accurately reflects the impact of this regimen on the general population of patients with advanced colorectal cancer.

A further difference between the studies is the duration of treatment and timing of response assessment. We first assessed patients after only four treatments (i.e. 8 weeks) and again after eight treatments, whereas some groups assessed patients after six and 12 treatments (De Gramont et al, 1988; Johnson et al, 1991). Early assessment may result in patients stopping treatment prematurely, before a response is noted. In particular, Hanna et al (1995) suggested that continued treatment in patients with stable disease may account for some of the differences between the studies. Indeed, patients in one study with a high response rate (Becouarn et al, 1995) received a mean of ten cycles. However, in other studies with high response rates, the average number of cycles given was five (Johnson et al, 1991; De Gramont et al, 1995). This compares with a median of five cycles given in the current study and four cycles in another with a low response rate (Hanna et al, 1995). It is unclear what proportion of patients with stable disease after four cycles of treatment may go on to respond if treatment is continued. In the current study, one-third of the responses were first noted only after eight cycles of treatment. Only 15% of our patients continued treatment beyond eight cycles and just one subsequently responded. Graf et al (1994) have suggested a survival advantage of 4 months for patients with stable disease on chemotherapy in their analysis of the relationship between response to chemotherapy and survival in advanced colorectal cancer. Of those patients who stopped at or before eight cycles of treatment, 54% had progressive disease and a further 9% stopped because of toxicity. The remaining third of patients who stopped had stable disease or a response but stopped chemotherapy either on the clinician's recommendation (11%) or for other reasons, most often patient request (22%). This suggests that it may be difficult to continue 2-weekly treatment beyond eight cycles in this relatively unselected group of patients under the care of a large number of different clinicians.

Although there were minor differences in toxicities between the two cohorts, this was not enough to account for the significant difference in dose reductions in cohort B compared with cohort A (41% and 17% respectively). Nevertheless, there was still a difference in median 5-FU dose per week of 970 mg $m^{-2}$ per week in cohort A and 1068 mg $m^{-2}$ per week in cohort B (an increase of 10% in 5-FU dose). On reviewing these dose reductions, most were the result of toxicities, recorded as minor by the clinician, that patients found to be unacceptable, e.g. persistent grade 1 mucositis or grade 2 hand–foot syndrome. This perhaps reflects more the realistic level of acceptable toxicity of palliative chemotherapy in this setting. We did, however, confirm the low incidence of grade 3 or above toxicity with this regimen found by others. The number of dose reductions and delays is only quoted in two of the other studies (Jodrell et al, 1994; Hanna et al, 1995).

The primary aim of this report is to evaluate the effect of 5-FU dose on treatment outcome. This study was the combination of two sequential phase II studies to determine outcome at 500 and 600 mg $m^{-2}$ of 5-FU. The response rate for each of these studies was performed using a group sequential triangular procedure. This involves examining the response rate in consecutive cohorts of patients (in this case ten or 20) and is a relatively novel approach to clinical evaluation of cancer treatment. In both cohorts, the sequential triangular test indicated closing each cohort after response results became evaluable for the first 60 evaluable patients (Figure 2). Because of rapid accrual and delays in obtaining response assessments, data were available only after additional patients had been recruited. This 'overshoot' was not described in a previous report of the use of the group sequential triangular test (Dieras et al, 1996). We were collecting response data on patients whose scans were performed over the entire West of Scotland, and this led to understandable delays. This practical issue of the commitment needed for the rapid collection of response data should be noted by other groups wishing to use this method of analysis. Nevertheless, using the sequential triangular test in our study, we have calculated that we entered 59% fewer evaluable patients than would have been required for the two equivalent phase II studies for the response rates and confidence intervals seen.

A formal comparison of the two 5-FU dose levels would optimally be made using a randomized study, whereas we report two consecutive phase II studies. When the results of cohort A became available, a randomized study was considered. However, some clinicians felt that, in view of the low response rate with 500 mg $m^{-2}$ 5-FU, and the possibility of a dose–response effect (Jodrell et al, 1994), they preferred to investigate the 600 mg $m^{-2}$ dose level in a further phase II study. Nevertheless, the two cohorts were well matched, although we did see significantly more patients with a raised alkaline phosphatase and fewer patients with performance status 0 in cohort B. After adjusting for prognostic factors, including alkaline phosphatase and performance status, there was a trend towards increased survival in cohort B compared with cohort A, with a death rate ratio of 0.77, but this was not significant (95% CI 0.54–1.11, $P$ = 0.16). It is disappointing that we found a very consistent low response rate of 18% and median survival of 7.8 months in both cohorts. Although dose reductions in cohort B meant that we achieved a dose increase of only 10%, rather than the 20% intended, we have no good evidence from this study to support increasing the dose of 5-FU from 500 to 600 mg $m^{-2}$. Indeed, taken with the results of our previous study (Jodrell et al, 1994), we have found a ceiling of 5-FU dose effect at 500 mg $m^{-2}$, with further dose increases difficult to administer in this group of patients.

Although response rates and survival using this 48-h bolus/infusion regimen may be lower in a general population than initially reported, in a randomized study (De Gramont et al, 1995) it did achieve a higher response rate than a monthly 5-day bolus 5-FU regimen (32.2% and 13.8% respectively, $P = 0.002$). However, even in that study, there was no significant difference in overall median survival between the two groups (61 and 58 weeks respectively) and a benefit of only 5 weeks in progression-free survival with the 48-h regimen (26.7 and 21.6 weeks respectively, $P = 0.007$). In this palliative setting with relatively minor differences between regimens, the fundamental issue is the quality of life of these patients. We collected no quality of life data on this group of patients, and there was no formal assessment of quality of life in any of the previous six studies on this regimen. However, Becouarn et al (1995) state that 74% of symptomatic patients had an improvement of their symptoms on the regimen. Likewise, Johnson et al (1991) stated that performance status rose in 60% of patients and 70% reported a subjective improvement in overall well-being with treatment. Hanna et al (1995) indicated that in their study 12% of the non-responding patients improved symptomatically during chemotherapy. These data suggest that with 44% of our patients having stable disease, we may have significantly underestimated the benefit of this regimen in terms of symptom control. It is important that future studies address the issue of quality of life, and this is a central part of the MRC trial of this regimen compared with continuous infusional 5-FU and Tomudex, which will also include cost benefit analysis.

In conclusion, we have used a novel trial methodology, the group sequential design, to investigate two dose levels of 5-FU given as a 48-h regimen. We found a response rate of 18% and a median survival of 7.8 months with this regimen in a relatively unselected group of patients with advanced colorectal carcinoma. These results may be explained in part by patient selection and treatment duration. These data suggest that increasing the dose intensity of this regimen is unlikely to improve outcome. Rather, it is important to compare this 48-h 5-FU schedule with other regimens in terms of response, survival and quality of life in prospective randomized trials before being accepted as routine clinical practice.

### ACKNOWLEDGEMENTS

We would like to thank the following participating consultants at the Beatson Oncology Centre who also entered patients into this study: D Bissett, AN Harnett, RD Jones, EJ Junor, SB Kaye, F MacBeth, JM Russell, WP Steward and RP Symonds.

### REFERENCES

Advanced Colorectal Cancer Meta-analysis Project (1992) Modulation of fluorouracil by leucovorin in patients with advanced colorectal cancer: evidence in terms of response rate. *J Clin Oncol* **10**: 896–903

Armitage P and Berry G (1987) Statistical Methods in Medical Research. 2nd edn. Blackwell Scientific Publications: Oxford

Becouarn YH, Brunet RC, Rouhier MLP, Bussieres EJ, Avril AR, Richaud PM and Dilhuydy JMA (1995) High dose folinic acid and continuous infusion for patients with advanced colorectal cancer. *Cancer* **76**: 1126–1131

Bellissant E, Beinchou J and Chastang C (1996) The group sequential triangular test for phase II cancer clinical trials. *Am J Clin Oncol* **19**: 422–430

De Gramont A, Krulik M, Cady J, Lagadec B, Maisani JE, Loiseau JP, Grange JD, Gonzalezcanall G, Demuynck B, Louvet C, Seroka J, Dray C and Debray J (1988) High dose folinic acid and 5-fluorouracil bolus and continuous infusion in advanced colorectal cancer. *Eur J Cancer* **24**: 1499–1503

De Gramont A, Bosset JF, Milan C, Rougier P, Bouché O, Etienne PL, Morvan F, Louvet C, Guillot T and Bedenne L (1995) A prospective randomized trial comparing 5FU bolus with low dose folinic acid (FUFOLld) and 5FU bolus plus continuous infusion with high dose colorectal cancer (abstract 455). *ASCO Proc*: **14**

Dieras V, Extra JM, Bellisant, Espie M, Morvan F, Pierga JY, Mignot L, Tresca P and Marty M (1996) Efficacy and tolerance of vinorelbine and fluorouracil combination as first line chemotherapy of advanced breast cancer: Results of a phase II study using a sequential group method. *J Clin Oncol* **14**: 3097–3104

Graf W, Pahlman L, Bergstrom R and Glimelius B (1994) The relationship between an objective response to chemotherapy and survival in advanced colorectal cancer. *Br J Cancer* **70**: 559–563

Hanna CL, McKinna FE, Williams LB, Morrey D, Adams M, Mason MD and Maughan TS (1995) High dose folinic acid and 5-fluorouracil bolus and continuous infusion in advanced colorectal cancer: poor response rate in unselected patients. *Br J Cancer* **72**: 774–776

Jodrell DI, Murray LS, Reed NS, Canney PA, Kaye SB and Cassidy J (1994) Bolus/infusional 5-fluorouracil and folinic acid for metastatic colorectal carcinoma: are suboptimal dosages being used in the UK? *Br J Cancer* **70**: 749–752

Johnson PWM, Thompson PI, Seymour MT, Deasy NP, Thuraisingham RC, Slevin ML and Wrigley PFM (1991) A less toxic regimen of 5-fluorouracil and high dose folinic acid for advanced gastrointestinal adenocarcinomas. *Br J Cancer* **64**: 603–605

Miller AB, Hoogstraten B, Staquet M and Winkler A (1981) Reporting results of cancer treatment. *Cancer* **47**: 207–214

Parmar MKB and Machin D (1995) *Survival Analysis: a Practical Approach*, pp. 26–33. John Wiley and Sons: Chichester

Peto J (1984) Calculation and interpretation of survival curves. In *Cancer Clinical Trials: Methods and Practice*, Buyse ME, Staquet MJ and Sylvester RJ. (eds), pp. 361–380. Oxford University Press: Oxford

Petrelli NJ (1995) It is time to stratify with prognostic factors for hepatic metastases. *J Clin Oncol* **13**: 2471–2476

Whitehead J (1982) The use of sequential probability ratio test for monitoring the percentage germination of accessions to seed banks. *Biometrics* **37**: 129–136

Whitehead J and Brunier H (1989) PEST 2.0. The PEST project; Department of Applied Statistics, University of Reading, UK

© Cancer Research Campaign 1998


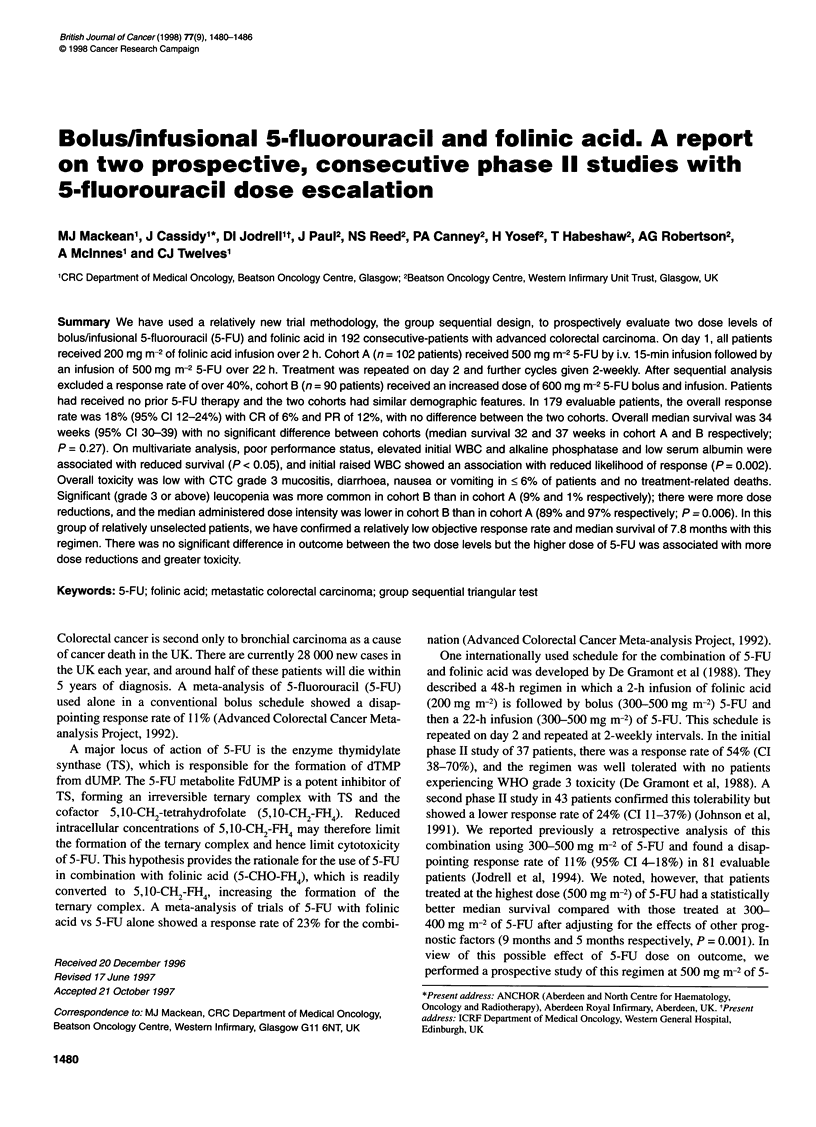

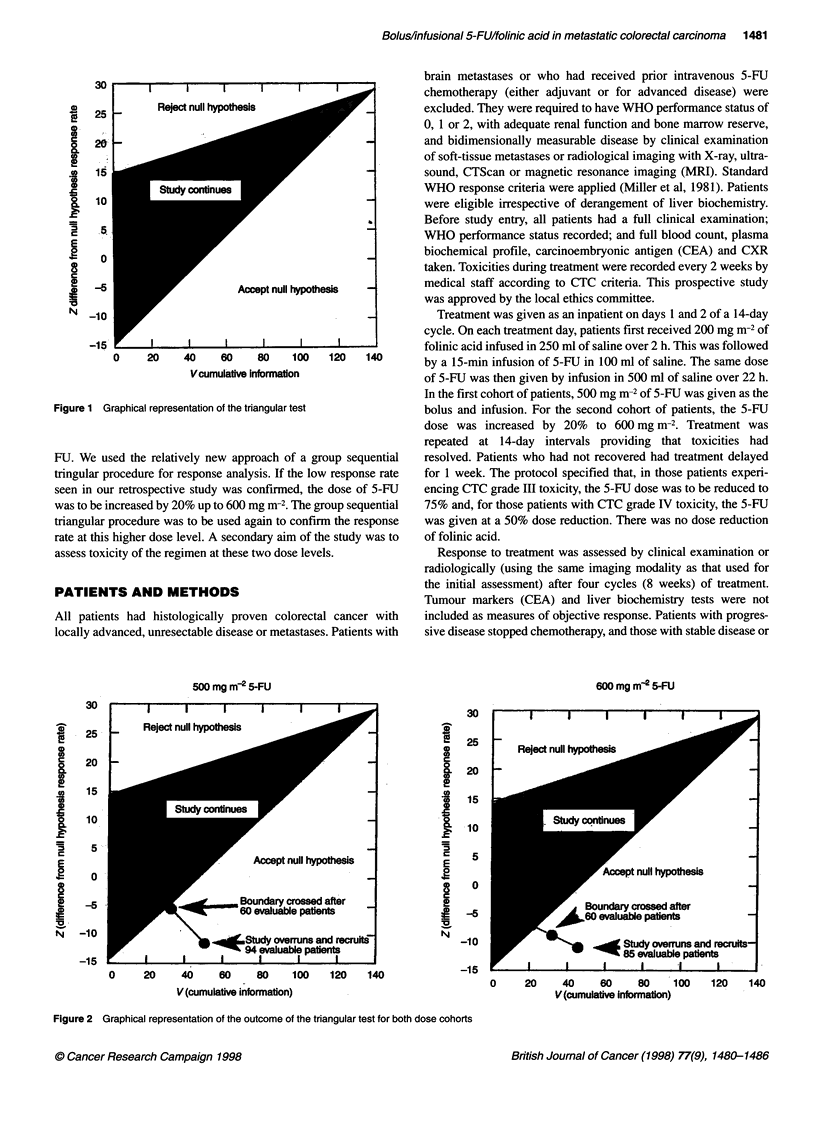

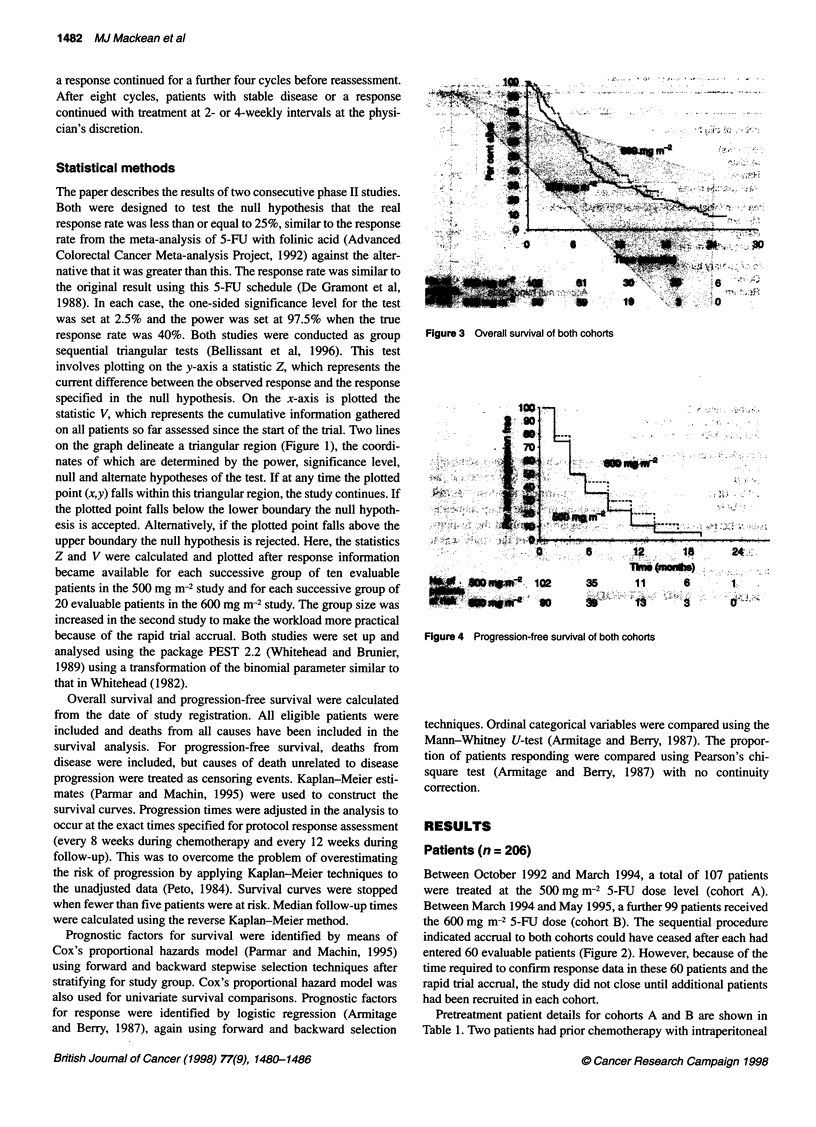

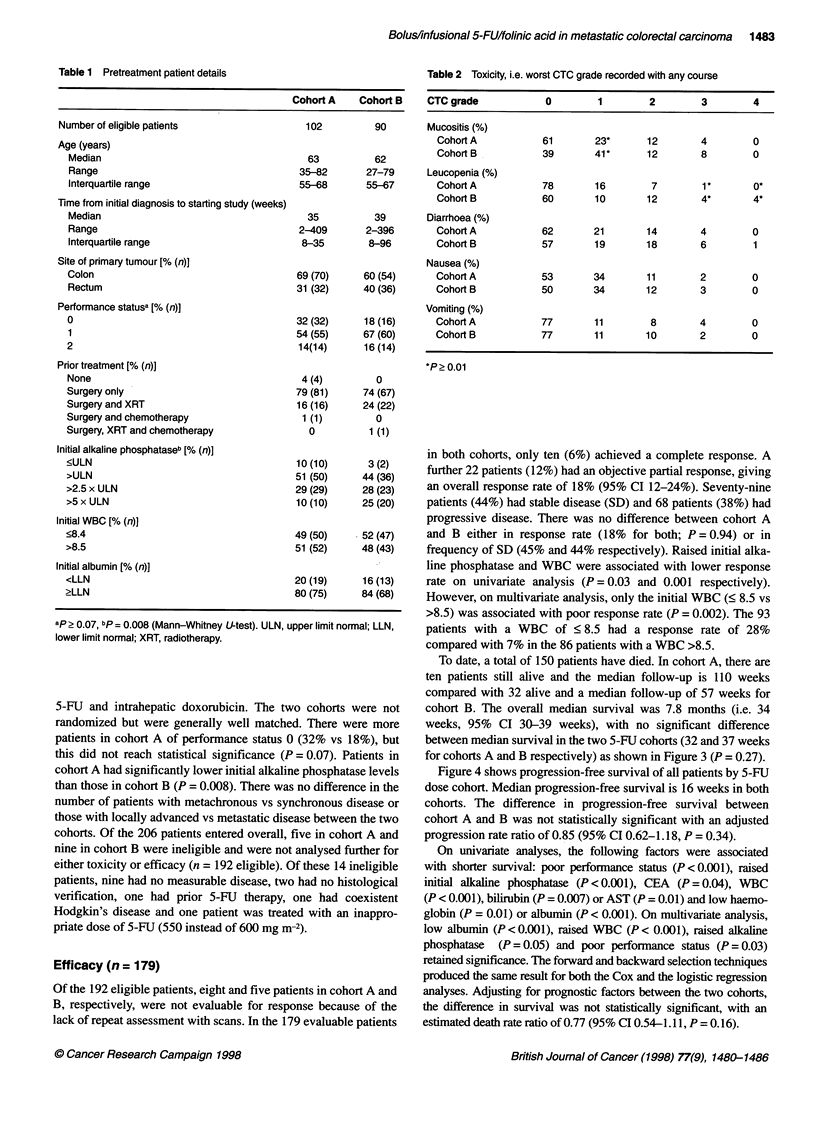

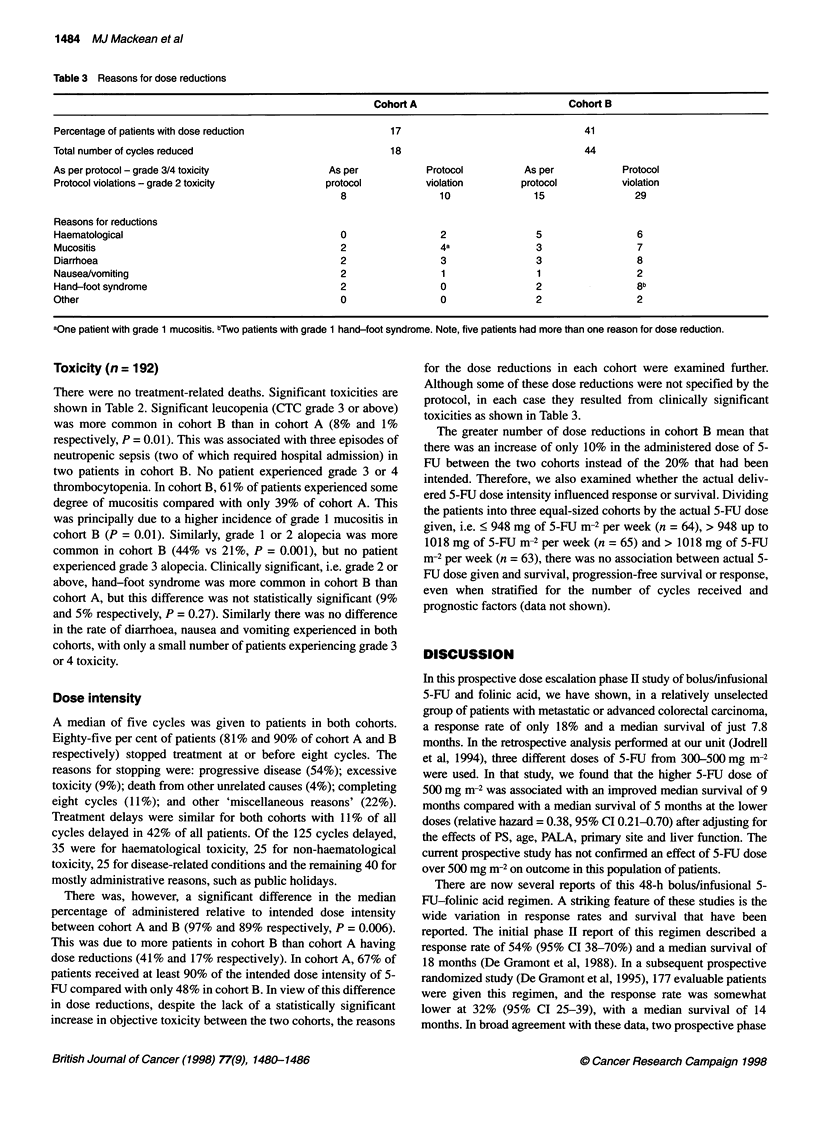

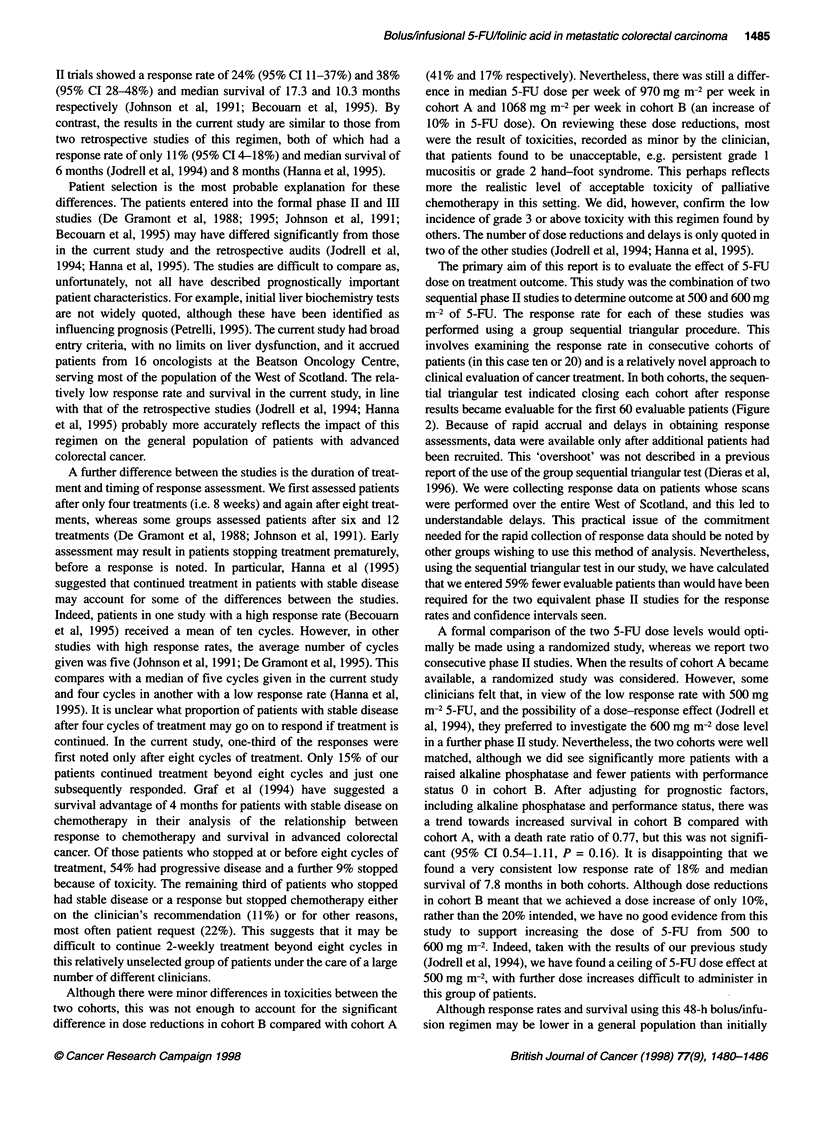

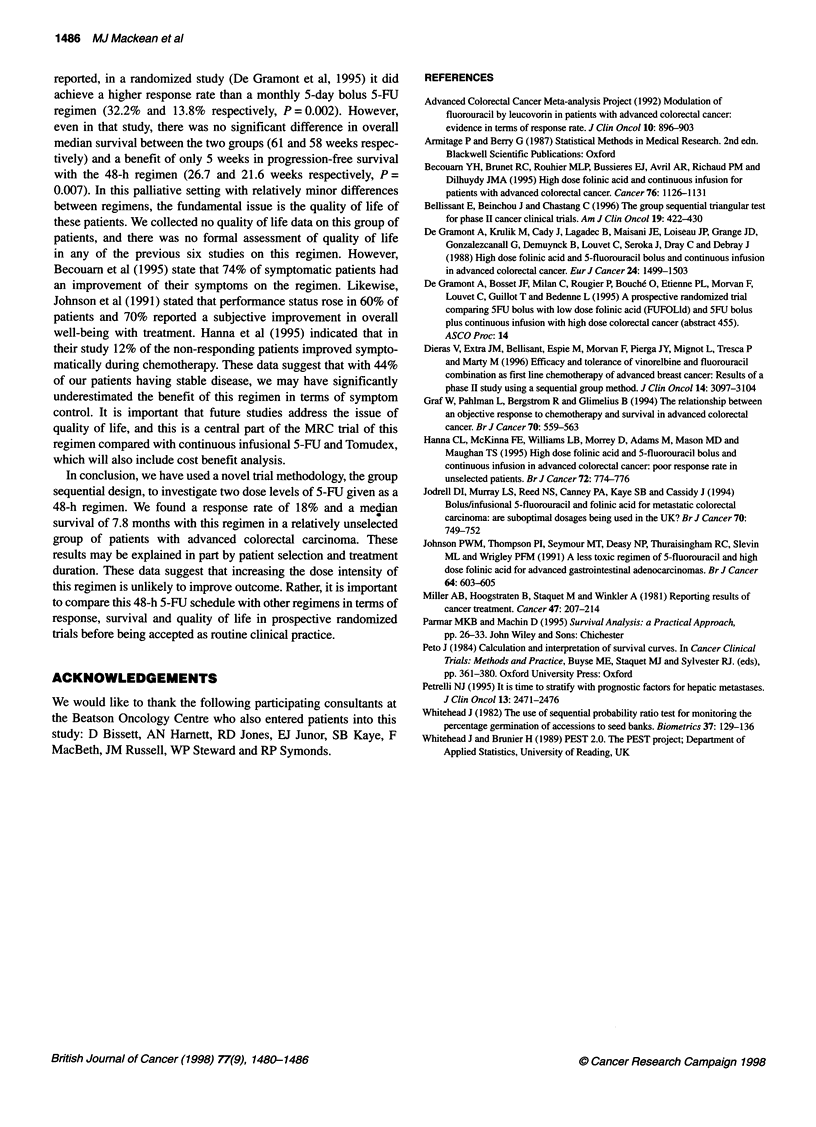


## References

[OCR_00888] Bellissant E., Bénichou J., Chastang C. (1996). The group sequential triangular test for phase II cancer clinical trials.. Am J Clin Oncol.

[OCR_00883] Bécouarn Y. H., Brunet R. C., Rouhier M. L., Bussières E. J., Avril A. R., Richaud P. M., Dilhuydy J. M. (1995). High dose folinic acid and 5-fluorouracil bolus and continuous infusion for patients with advanced colorectal cancer.. Cancer.

[OCR_00892] De Gramont A., Krulik M., Cady J., Lagadec B., Maisani J. E., Loiseau J. P., Grange J. D., Gonzalez-Canali G., Demuynck B., Louvet C. (1988). High-dose folinic acid and 5-fluorouracil bolus and continuous infusion in advanced colorectal cancer.. Eur J Cancer Clin Oncol.

[OCR_00907] Dieras V., Extra J. M., Bellissant E., Espie M., Morvan F., Pierga J. Y., Mignot L., Tresca P., Marty M. (1996). Efficacy and tolerance of vinorelbine and fluorouracil combination as first-line chemotherapy of advanced breast cancer: results of a phase II study using a sequential group method.. J Clin Oncol.

[OCR_00914] Graf W., Påhlman L., Bergström R., Glimelius B. (1994). The relationship between an objective response to chemotherapy and survival in advanced colorectal cancer.. Br J Cancer.

[OCR_00919] Hanna C. L., McKinna F. E., Williams L. B., Morrey D., Adams M., Mason M. D., Maughan T. S. (1995). High-dose folinic acid and 5-fluorouracil bolus and continuous infusion in advanced colorectal cancer: poor response rate in unselected patients.. Br J Cancer.

[OCR_00925] Jodrell D. I., Murray L. S., Reed N. S., Canney P. A., Kaye S. B., Cassidy J. (1994). Bolus/infusional 5-fluorouracil and folinic acid for metastatic colorectal carcinoma: are suboptimal dosages being used in the UK?. Br J Cancer.

[OCR_00932] Johnson P. W., Thompson P. I., Seymour M. T., Deasy N. P., Thuraisingham R. C., Slevin M. L., Wrigley P. F. (1991). A less toxic regimen of 5-fluorouracil and high-dose folinic acid for advanced gastrointestinal adenocarcinomas.. Br J Cancer.

[OCR_00938] Miller A. B., Hoogstraten B., Staquet M., Winkler A. (1981). Reporting results of cancer treatment.. Cancer.

[OCR_00951] Petrelli N. J. (1995). It is time to stratify with prognostic factors for hepatic metastases.. J Clin Oncol.

